# Solid state fermentation: A strategy for wheat bran supplemented corn stover valorization with *Pleurotus* species

**DOI:** 10.3934/microbiol.2025011

**Published:** 2025-03-17

**Authors:** Michael Wuaku, Omoanghe S. Isikhuemhen, Peter A. Dele, Chika C. Anotaenwere, Ahmed E. Kholif, Oludotun O. Adelusi, Joel O. Alabi, Deborah O. Okedoyin, DeAndrea Gray, Kelechi A. Ike, Olatunde A. Oderinwale, Jorge A. Villarreal-González, Nkese S. Udombang, Judith O. Enemudo, Nathan Holt, Brandon G. Essick, Felicia N. Anike, Lauren Mayo, Uchenna Y. Anele

**Affiliations:** 1 Department of Animal Sciences, North Carolina Agricultural and Technical State University, Greensboro, NC 27411, USA; 2 Mushroom Biology & Fungal Biotechnology Laboratory, Department of Natural Resources and Environmental Design, North Carolina A&T State University, Greensboro, NC 27407, USA; 3 Department of Pasture and Range Management, Federal University of Agriculture, Abeokuta, Ogun State 110001, Nigeria; 4 Dairy Science Department, National Research Centre, 33 Bohouth St. Dokki, Giza, Egypt

**Keywords:** solid-state fermentation, *Pleurotus* spp., *in vitro* digestibility, feeding value, corn stover

## Abstract

This study compared the potential of three white-rot fungi (*Pleurotus* spp.) to enhance the nutritional value of corn stover as a feed resource for ruminants. A mixture of shredded corn stover and wheat bran (ratio 9:1) was moisturized (65%), loaded into polypropylene bags, and sterilized at 121 °C for 1 h. Four replicate bags were each inoculated with *P. ostreatus* (isolates P1 and P3) and *P. pulmonarius* (isolate P2) and incubated at 25 °C for 0, 2, 4, 6, and 8 weeks. After inoculation and incubation of the corn stover, the resultant substrates and rumen fluid obtained from three ruminally cannulated beef cows were investigated using an *in vitro* batch culture study, designed as a 3 × 5 factorial with six replicates. Results revealed a significant (p < 0.001) effect on dry matter digestibility (DMD), with the highest DMD observed at 8 weeks for all *Pleurotus* isolates tested. The best (p < 0.001) performance was seen in corn stover treated with P2 at weeks 6 and 8. Additionally, P1 at 0 and 6 weeks had the lowest ash and highest (p < 0.001) organic matter (OM) concentrations, respectively, compared to P2 at 8 weeks, which had the highest ash and the lowest OM concentrations. The highest (p = 0.011) crude protein (CP) content was recorded in P1 at week 8, while P1 at week 0 had the lowest CP content. Compared to untreated corn stover, higher (p < 0.001) acid detergent fiber digestibility was recorded in corn stover treated with P2 at 8 weeks, while higher (p < 0.001) neutral detergent fiber digestibility was observed in P3 at 2 weeks. *Pleurotus* strains and incubation periods affected microbial mass production (p < 0.001), with minimal effects on total and individual volatile fatty acids. However, P3 at 2 weeks increased (p = 0.035) acetate and decreased (p = 0.001) propionate proportions. The results indicate that different isolates affected corn stover differently, but in general, all isolates improved the nutritional value of corn stover. *P. pulmonarius* had the highest DMD and lowest fiber content among the isolates tested and improved energy and nutrient utilization.

## 1. Introduction

The use of white rot fungi (WRF) in solid-state fermentation (SSF) to increase the nutritional values of lignocellulosic biomass is a subject of great interest due to the increasing demand for alternative and sustainable ruminant feed materials. Corn stover treated with WRF during SSF has higher fermentable sugars, enhanced saccharification properties, modified lignin, and improved nutritional value, making it a better ruminant feed [1]–[4]. Kholif et al. [5] demonstrated the WRF conversion of rice straw to energy-rich cattle feed. Also, Akinfemi et al. [6] used *Pleurotus ostreatus* and *P. pulmonarius* to improve the conversion of sorghum stover and reported enhanced potential in ruminant nutrition. This highlights the need for either full or partial lignin degradation from the lignocellulosic complex [7]. Saminathan [8] summarized the use of treated oil palm fronds as an enhanced feed source for ruminants, emphasizing the importance of lignin degradation to improve the nutritional value of oil palm fronds. The ubiquitous use of straw as a roughage source in cattle diets was highlighted by Wang et al. [1], who investigated the use of four different *Pleurotus* spp. to enhance the nutritional content of fermented corn stover as ruminant feed. Kholif et al. [4] demonstrated that treating date palm leaves with *P. ostreatus* for 45 days reduced the fiber fractions, total gas, CH_4_, and CO_2_ production, as well as the rate of CH_4_ and CO_2_ production. Olagunju et al. [9] reported an increase in crude protein (CP), ash, total volatile fatty acids (VFA), propionate, microbial mass, *in vitro* apparent degradable dry matter (IVADDM), *in vitro* truly degradable dry matter (IVTDDM), and dry matter digestibility (DMD) values on SSF corn stover. Recently, Anotaenwere et al. [10] treated corn stover with *P. ostreatus* and ensiled it for 0, 21, 42, and 64 days. The ensiled material was then included in diets at levels of 10%, 20%, 30%, 40%, and 50%. Their results showed that higher levels of spent mushroom substrate in the diets increased DM, neutral detergent fiber (NDF), and acid detergent fiber (ADF) concentrations while decreasing CP and cellulose (CEL) levels. Additionally, they reported that diets with higher amounts of spent mushroom substrate also led to a decrease in gas production (GP), CH_4_, and CO_2_ production, while improving the degradability of DM, NDF, ADF, and acid detergent lignin (ADL). This validated the possibility of converting corn stover into animal feed with enhanced nutritional value. As reported in other studies [11],[12], WRF play a crucial role in the utilization of lignocellulosic materials because they break down lignin, CEL, and hemicellulose (HEM), which are the three main components of plant cell walls [4]. WRF secrete health-promoting bioactive chemicals and utilize peroxidases and laccases, the two essential enzymes involved in lignin degradation [13]. They also use these enzymes to achieve lignin depolymerization [12]. Before being fed to cattle, mushrooms grown on wheat straw allow fungal enzymes to break down lignin in the straw [14],[15]. Additionally, mushrooms can secrete bioactive compounds that modulate health, such as proteins, lectins, and glycopolysaccharides [16]–[18]. These substances may improve the health of cattle [19].

There is a paucity of information on how different *Pleurotus* spp. isolates affect the feeding value and nutrient digestibility of corn stover after SSF. This study advances previous research by systematically comparing the effects of three *Pleurotus* isolates, over multiple incubation periods, addressing the limitation of prior studies that focused on single fungal strains or fixed incubation durations. The current study goes beyond previous studies that primarily examined chemical composition by also evaluating microbial mass production and rumen fermentation of the substrates. By identifying the most effective fungal species and incubation periods for optimizing corn stover as a ruminant feed, this study offers practical recommendations for sustainable livestock production. It contributes to the efficient use of agricultural byproducts and helps reduce reliance on conventional feed resources. Thus, the objective of this study was to investigate how two isolates of *P. ostreatus* (isolates P1 and P3) and *P. pulmonarius* (isolate P2) can improve the feed value and nutrient content of corn stover using an *in vitro* batch culture technique.

## 2. Materials and methods

### 2.1. Solid-state fermentation

In this study, the selection of *Pleurotus* isolates (P1, P2, and P3) was based on their distinct physiological and ecological traits, which influence their ability to degrade lignocellulosic materials. P1 and P2 are well-established commercial *P. ostreatus* strains widely used in the mushroom industry, recognized for their efficient lignin-degrading enzyme systems, making them ideal candidates for improving the digestibility of fibrous feed resources. *P. pulmonarius* (P2) was specifically chosen for its high-temperature tolerance, which is advantageous for fungal fermentation under varying environmental conditions, ensuring its practical applicability in diverse feed processing settings. In contrast, P3 is a wild isolate from Mount Aire, North Carolina, selected to explore the potential of naturally adapted strains that may possess unique enzymatic capabilities and fiber-degrading efficiency. By comparing commercial and wild isolates, this study aimed to identify the most effective fungal treatment for enhancing the nutritional value of corn stover while considering both industrial feasibility and the adaptability of strains to different environmental conditions.

Spawn was prepared following a modified method previously reported [9]. Sorghum grains (*Sorghum bicolor*) were briefly soaked in water for 12 h and drained before use. Twelve replicate polypropylene bags (580 × 490 mm) weighing 1 kg were prepared from the sorghum grains and sterilized (121 °C, 15 psi for 1 h). After cooling, four bags each were inoculated with 5-day-old pure cultures of P1, P2, or P3, pre-cultivated on PDA medium plates (100 cm). The inoculated bags were sealed and incubated at 25 °C for 14 days before use in substrate inoculation.

Corn stover obtained from North Carolina Agricultural and Technical State University Farm was shredded into 2–5 cm pieces and soaked in water for 12 h, drained of water, mixed with wheat bran (10%), and loaded into micropore-fitted polypropylene cultivation bags. Wheat bran was added to the corn stover substrate as a readily fermentable co-substrate to support fungal growth and enzymatic activity. Its rich content of essential nutrients and easily degradable carbohydrates provides an initial energy source for the *Pleurotus* spp., facilitating the establishment and proliferation of fungal mycelium. Each bag weighed 2.5 kg. The bags were sterilized at 121 °C and 15 psi for 1 h. After cooling overnight, substrate bags were moved to a laminar flow hood. Each corn stover substrate bag (2.5 kg) was inoculated with the spawn (250 g), mixed to distribute the spawn throughout the substrate bag, and sealed. All inoculated bags, 30 each per strain, were transferred to the incubation room for the SSF process, which lasted for 2, 4, 6, and 8 weeks. The selection of these incubation periods aimed at capturing the dynamic changes in fungal degradation of corn stover over time, while considering the practical applicability for feed processing. These time points were chosen to monitor the progressive breakdown of lignocellulosic components, assess nutrient enhancement, and identify the optimal duration for maximizing digestibility and microbial accessibility. The initial (week 0) sampling served as a baseline to compare untreated and treated corn stover, while the early (weeks 2 and 4) time points allowed for the evaluation of rapid enzymatic activity and initial structural modifications. The later incubation periods (weeks 6 and 8) were included to determine whether prolonged fungal treatment would further enhance nutrient availability or reach a plateau, where additional incubation would offer diminishing returns.

The control treatment was inoculated, then immediately dried and stored for use in further studies. At each sampling time, five bags per treatment were taken out and dried at 55 °C until they were at a constant weight (usually 4–5 days). The dried treated corn stover substrates (five per treatment) and control were separately milled to pass through a 1 mm mesh and then homogenized before being used in chemical analysis and batch culture experiments.

### 2.2. Animal care and feeding

The Institutional Animal Care and Use Committee (IACUC) at the North Carolina Agricultural and Technical State University, Greensboro, authorized all environmental procedures and animal use (IACUC-approved protocol LA21-009) for the study at the University Beef Research and Training Facility (BRTF, NCAT University Farm, Greensboro, NC, USA). Cannulated cows were maintained according to the standard management procedures.

### 2.3. Experimental design

The experimental design was a 3 × 5 factorial design with three WRF strains and five incubation periods. All treatments were analyzed for chemical composition and evaluated in an *in vitro* batch culture experiment at 6 and 24 h with six replicates per treatment per incubation period. The study was conducted in two separate runs across different weeks to improve the reliability and validity of the results.

### 2.4. Chemical analysis

The proximate contents of treated corn stover were determined according to the standard protocols [20]. For DM determination, samples were weighed, oven-dried for 48 h at 55 °C, and weighed after drying. Weighed samples were burned to determine the ash content for 3 h at 550 °C in a muffle furnace (model BF51728C-1; Thermo Fisher Scientific, Asheville, NC, USA). The C, H, and N analyses were conducted according to the Classical Pregl-Dumas method using a PerkinElmer CHN/O analyzer (model 2400 Series II; PerkinElmer Inc., Waltham, MA, USA). The CP was then calculated by multiplying the N concentration by 6.25. Crude fat was extracted using petroleum ether in an Ankom XT15 fat extractor system (Ankom Technology Corp., Fairport, NY, USA). The NDF was analyzed using the filter bag technique method [21] with sodium sulfite and a heat-stable alpha-amylase; subsequently, ADF was analyzed using an Ankom 200 fiber analyzer (Ankom Technology; Macedon, NY, USA). The ADL was determined by soaking in 72% sulfuric acid (H_2_SO_4_) in a beaker for 3 h. HEM content was calculated from the difference between NDF and ADF, and CEL content from the difference between ADF and ADL. Non-structural carbohydrate (NSC = 100 − NDF − CP − EE − ash), and organic matter (OM = 100 − ash) concentrations were calculated.

### 2.5. Sample preparation for the in vitro assay

Approximately, 0.5 ± 0.05 g of samples (n = 9 replicates per treatment) were weighed with an analytical scale (model VWR-224AC; VWR International, Radnor, PA, USA) directly into labeled 100 mL serum bottles (Cat# 223747; Wheaton Science Products, Millville, NJ, USA) to estimate microbial mass. Six additional replicates per treatment were weighed into Ankom bags (F57; Ankom Technology Corp., Macedon, NY, USA), sealed with a heat impulse sealer (Model # AIE-200HR, California, USA), and put into the labeled 100 mL serum bottles to determine nutrient digestibility.

### 2.6. In vitro batch culture

Three rumen-cannulated, Black Angus cows (average body weight of 550 ± 10 kg) served as rumen inoculum donors. The cows were maintained predominantly on forage (99% hay and grass) and 1% mineral supplement with ad libitum access to pasture and water. The rumen fluid (RF) was collected 4 h post-meal from the rumen's dorsal, ventral, and cranial regions and strained through four layers of cheesecloth to filter out the solid digesta. Then, the rumen inoculum was brought in a prewarmed, insulated Thermoflask to the Ruminant Nutrition laboratory. The pH of the pooled RF was measured using a benchtop pH meter (model B10P; VWR International, Radnor, PA, USA).

The *in vitro* batch culture followed the procedures described by Anele et al. [22] with a brief modification. The bags were placed in 100 mL serum bottles containing 45 mL of McDougall's buffer, which had been CO_2_-gassed and prewarmed at 39 °C in a water bath (model JAB18; Grant Instruments Ltd., Cambridge, UK). Then, 15 mL aliquots of strained RF, anaerobically flushed with CO_2_ and maintained at 39 °C, were added. Rubber stoppers (20 mm) and metal caps (20 mm) (Wheaton Science Products, Millville, New Jersey, USA) were used to cap and crimp the bottles. The *in vitro* batch fermentation was carried out using an orbital shaker running at 125 rpm in a reach-in incubator (VWR International, Radnor, PA, USA) at 39 °C. For the digestibility study, six blanks were used to correct for background GP. The blanks were made in accordance with the experimental design for each time period. The *in vitro* batch experiments were carried out twice, on different successive weeks, using RF from the same animals.

Gas pressure for each serum bottle was measured at each time interval to determine the amount of gas produced by inserting a hypodermic gauge needle (BD 0.7 × 40 mm, Cat# 301000) attached to a manometer (model 33500-086; VWR International, Radnor, PA, USA). After the 6 and 24 h periods, serum bottles were decapped using a decapping plier (Cat# C4020-101; Thermo Scientific, Rockwood, TN, USA). The Ankom bags were cleaned with cold water and oven-dried for 48 h at 55 °C. Thereafter, oven-dried bags were put in a desiccator for 20 min and then weighed to determine the dry matter disappearance (DMD). Thereafter, NDF, ADF, and ADL digestibility were determined as described in the chemical analysis.

### 2.7. Microbial mass

Microbial mass was determined according to the modified Blümmel and Lebzien protocol previously outlined [23]. The digesta content from the serum bottles was transferred into pre-weighed 50 mL Eppendorf tubes (Thermo Scientific Nalgene Products; Rochester, NY, USA) and centrifuged using a Thermo Fisher Scientific centrifuge (model SORVALL RC-6 plus; Thermo Fisher Scientific Inc., Asheville, NC, USA) at 15,000 × g for 15 min at 4 °C. Blank samples were included as a correction factor in the experimental process. The pellets after centrifuging were frozen at −20 °C for 24 h. The frozen samples were freeze-dried for 96 h using a BUCHI freeze dryer (Lyovapor L-200, BUCHI, New Castle, DE, USA). After freeze-drying, tubes were reweighed, and the microbial mass was calculated using the following equation:

Microbial mass = [Feed DM incubated – (Pellet DM − Blank pellet DM)]/Feed DM incubated.

Thereafter, IVADDM and IVTDDM were estimated. The partitioning factor (PF_24_) was computed as the ratio of milligram of substrate degraded per milliliter and gas generated at 24 h [24].

### 2.8. Volatile fatty acid

For VFA concentration, 15 mL of digesta content from each serum bottle was sampled into a vial containing 3 mL of 25% metaphosphoric-crotonic acid and frozen at −20 °C until required for analysis. Samples were defrosted and then centrifuged at 10,000 × g for 15 min at 4 °C using a Thermo Fisher Scientific centrifuge (model Sorvall X4R Pro-MD; Thermo Electron LED GmbH, Osterode, Germany). The VFA profile was quantified using automated gas chromatography (Agilent 7890B GC system/5977B GC-MSD/7693 autosampler, Agilent Technologies, Santa Clara, CA, USA). Using ethyl acetate as an internal standard, VFA were separated with a capillary column (Zebron ZB-FFP, Phenomenex Inc., Torrance, CA, USA) for acetate (C_2_), propionate (C_3_), butyrate (C_4_), isobutyrate (iso-C_4_), valerate (C_5_), and isovalerate (iso-C_5_) concentration determination. The total VFA and C_2_:C_3_ ratio were estimated as previously described [9].

### 2.9. Estimated parameters

The equations given by Fonnesbeck et al. [25] and Undersander et al. [26] and adapted from Olagunju et al. [9] were used to calculate the estimated parameters: Dry matter intake (DMI) = 120/NDF, dry matter digestibility (DDM) = 88.9 − (0.779 × ADF), relative forage value (RFV) = (DDM × DMI) × 0.775, relative forage quality (RFQ) = (DMI × TDN)/1.23, total digestible nutrient (TDN, %) = 104.97 − (1.302 × ADF), digestible CP (DCP) = (0.916 × CP) − 3.09, gross energy (GE, Kcal/g) = (CP × 0.056) + (EE × 0.094) + (100 − CP − Ash − EE) × 0.042.

### 2.10. Statistical analysis

Data were analyzed as repeated measures using PROC MIXED procedure (SAS 9.4 version; SAS Institute Inc., Cary, NC, USA). The fixed effect of treatment and repeated measures (sampling day) were considered by the MIXED model. Different covariance structures were explored for the repeated measures, and the ultimate decision was made based on low values from Akaike's information criteria (AIC), which were compared using Tukey's test. The model used was: Y_ijk_ = µ + T_i_ + P_j_ + (TP)_ij_ + e_ijk_, where Y_ijk_ is each individual observation for a given variable, µ is the overall mean, T_i_ is the strain effect, P_j_ is the incubation period effect, (TP)_ij_ is the interaction between strain and incubation period, and e_ijk_ is the residual error. Polynomial (linear and quadratic) contrasts (adjusted for the equal spacing of incubation weeks) were used to examine duration responses to increasing the incubation weeks. Significance was declared at p < 0.05.

## 3. Results

### 3.1. Chemical composition

Table 1 shows the chemical composition of all substrates treated with *Pleurotus* spp. isolates and incubation period. The strain × week interaction significantly (p < 0.05) affected all nutrient contents, except for EE. Additionally, the fungi strain influenced (p < 0.001) all nutrient values, except for CP. The incubation period had a linear effect (p < 0.001) on nutrient contents. The highest (p < 0.001) DM concentrations were observed with P3 across all incubation periods, while the lowest DM content was observed in P1 at week 0. Furthermore, P1 at weeks 0 and 6 of SSF had the lowest ash and highest OM (p < 0.001) concentrations, respectively, compared to P2 at week 8, which had the highest ash and lowest OM concentrations. The highest (p = 0.011) CP was recorded in P1 at week 8, whereas P1 at week 0 had the lowest CP value. The highest NSC content (p < 0.001) was observed in P2 at week 6, while P3 at week 0 had the lowest content. The highest NDF and ADF were observed in P2 at week 0, and P3 at week 2 of SSF, respectively, while the lowest NDF and ADF were observed with P1 at week 8. P2 at week 8 of SSF had the highest (p < 0.001) ADL and lowest HEM concentrations. The highest (p < 0.001) CEL value was observed in P3 at week 2, while the lowest value was observed in P1 at week 8.

### 3.2. In vitro gas production and DM digestibility

A significant interaction (p < 0.01) between fungi strain and incubation period was observed for GP, undegraded residuals, IVADDM, IVTDDM, PF_24_, and MM (Table 2). Additionally, the fungal strain significantly (p < 0.01) influenced all variables, except MM, while incubation period had a linear effect (p < 0.001) on all variables, except PF_24_. The highest GP was recorded in P2 at week 6, while the lowest GP was observed in P3 at week 4. Higher undegraded residuals were observed in P3 at week 2, while P2 at week 6 had the lowest residual values. Higher (p < 0.001) IVADDM and IVTDDM values were noted for P2 at weeks 6 and 8, respectively. P3 at week 4 had the highest PF_24_ (p = 0.003) compared to the lower PF_24_ values observed in P1 at weeks 4 and 6. Higher (p = 0.01) MM was observed for P3 at week 8 when compared with P2 at week 8.

**Table 1.** Chemical composition (% DM) of corn stover treated with *Pleurotus ostreatus* P1 and P3 isolates and *P*. *pulmonarius* P2 isolate.

Strain	Week	DM	OM	Ash	CP	EE	NSC	NDF	ADF	ADL	HEM	CEL
P1	0	94.9^i^	94.9^a^	5.06^f^	4.44^c^	2.31	7.7^e^	81.2^ab^	53.9^bc^	10.2^ef^	27.3^a^	43.7^ab^
	2	95.3^gh^	94.1^bc^	5.93^e^	5.67^bc^	2.89	17.0^bc^	68.5^fg^	49.4^d^	10.5^de^	19.2^de^	38.8^efg^
	4	95.6^fg^	91.8^e^	8.17^c^	6.44^ab^	1.16	17.7	66.5^h^	48.9^d^	11.7^bcd^	17.6^f^	37.2^g^
	6	95.1^hi^	95.0^a^	4.97^f^	5.79^bc^	3.02	18.9^b^	67.3^gh^	49.3^d^	6.85^g^	18.0^ef^	42.5^bc^
	8	95.5^fg^	89.7^f^	10.3^b^	7.38^a^	2.25	18.8^b^	61.3^j^	44.8^e^	11.2^cde^	16.4^f^	33.7^h^
P2	0	96.2^d^	93.3^d^	6.66^d^	4.65^c^	2.80	4.0^f^	81.9^a^	54.7^b^	12.8^ab^	27.1^a^	41.9^bcd^
	2	95.9^e^	94.7^ab^	5.29^ef^	5.23^bc^	1.76	13.1^d^	75.2^c^	52.6^c^	11.7^bcd^	22.6^c^	41.0^cd^
	4	96.4^cd^	93.4^cd^	6.60^d^	5.94^abc^	1.45	18.7^b^	67.3^gh^	49.7^d^	9.14^f^	17.6^f^	40.6^de^
	6	95.7^ef^	94.7^ab^	5.28^ef^	5.69^bc^	2.36	22.6^a^	64.1^i^	49.4^d^	7.33^g^	14.7^g^	42.1^bcd^
	8	96.7^c^	85.9^g^	14.1^a^	6.54^ab^	2.85	12.1^d^	64.3^i^	52.5^c^	14.0^a^	11.9^h^	38.5^fg^
P3	0	97.0^b^	92.4^e^	7.56^c^	5.85^abc^	2.17	4.5^f^	80.8^b^	54.8^b^	12.3^bc^	25.5^b^	42.4^bc^
	2	97.6^a^	94.5^ab^	5.54^ef^	6.38^ab^	2.17	5.2^ef^	81.4^ab^	57.3^a^	12.1^bc^	24.1^b^	45.2^a^
	4	97.4^a^	94.6^ab^	5.38^ef^	6.38^ab^	2.57	14.6^cd^	71.1^d^	54.1^bc^	11.3^cde^	17.0^f^	42.9^b^
	6	97.4^a^	94.5^ab^	5.52^ef^	6.31^ab^	1.66	16.5^bc^	70.0^de^	53.3^bc^	10.6^de^	16.8^f^	42.7^bc^
	8	97.6^a^	94.1^bc^	5.89^e^	6.21^abc^	2.78	16.3^bc^	68.8^ef^	48.3^d^	9.14^f^	20.5^d^	39.2^ef^
SEM		0.14	0.14	0.365	0.146	0.162	0.59	1.02	0.49	0.300	0.68	0.45
p-value												
Strain		<0.001	<0.001	<0.001	0.118	<0.001	<0.001	<0.001	<0.001	<0.001	<0.001	<0.001
Week												
Linear		<0.001	<0.001	<0.001	<0.001	<0.001	<0.001	<0.001	<0.001	<0.001	<0.001	<0.001
Quadratic		0.543	<0.001	<0.001	0.195	<0.001	<0.001	<0.001	0.583	<0.001	<0.001	<0.001
Strain × Week		<0.001	<0.001	<0.001	0.011	0.352	<0.001	<0.001	<0.001	<0.001	<0.001	<0.001

Means in the same column with different superscripts differ at p < 0.05. SEM, standard error of the mean; DM, dry matter; OM, organic matter; CP, crude protein; EE, ether extract; NSC, non-structural carbohydrate; NDF, neutral detergent fiber; ADF, acid detergent fiber; ADL, acid detergent lignin; HEM, hemicellulose; CEL, cellulose.

**Table 2.** Gas production, undegraded residuals, *in vitro* DM digestibility, partitioning factor, and microbial mass production efficiency of corn stover treated with *Pleurotus ostreatus* P1 and P3 isolates and *P*. *pulmonarius* P2 isolate.

Strain	Week	Gas, mL/g DM	Undegraded residuals, %	IVADDM, %	IVTDDM, %	PF_24_	MM, g/kg DM
P1	0	61.0^cdef^	30.8^c^	30.7^de^	35.9^g^	3.13^abcd^	0.025^bcd^
	2	64.7^bcdef^	29.3^d^	30.4^de^	39.4^ef^	3.12^abcd^	0.044^abc^
	4	78.2^abc^	28.2^de^	32.9^cd^	41.4^cde^	2.61^d^	0.041^abc^
	6	70.6^bcde^	31.1^c^	30.4^de^	35.9^g^	2.56^d^	0.026^abcd^
	8	73.1^bcd^	27.8^e^	37.2^b^	42.7^bcd^	2.89^bcd^	0.027^abcd^
P2	0	67.7^bcde^	33.5^ab^	31.3^cde^	31.8^i^	2.42^d^	0.027^abcd^
	2	56.4^def^	33.3^ab^	22.7^f^	31.0^i^	2.67^cd^	0.040^abc^
	4	78.8^abc^	27.4^e^	35.3^bc^	43.8^bc^	2.80^bcd^	0.042^abc^
	6	92.1^a^	24.4^f^	44.6^a^	49.7^a^	2.65^cd^	0.025^bcd^
	8	80.3^ab^	26.8^e^	45.0^a^	45.4^b^	2.91^bcd^	0.009^d^
P3	0	57.7^def^	33.1^ab^	29.4^de^	32.8^hi^	3.00^bcd^	0.020^cd^
	2	53.2^ef^	34.5^a^	28.9^de^	30.7^i^	2.90^bcd^	0.028^abcd^
	4	46.3^f^	32.3^bc^	27.9^e^	34.9^gh^	3.76^a^	0.035^abc^
	6	55.2^def^	31.1^c^	28.4^de^	37.3^fg^	3.39^abc^	0.044^ab^
	8	59.1^def^	29.4^d^	31.0^cde^	40.8^de^	3.48^ab^	0.048^a^
SEM		1.883	0.30	0.70	0.60	0.066	0.0020
p-value							
Strain		<0.001	<0.001	<0.001	<0.001	0.002	0.351
Week							
Linear		0.004	<0.001	<0.001	<0.001	0.284	<0.001
Quadratic		0.976	0.269	0.009	0.397	0.962	0.450
Strain × Week		<0.001	<0.001	<0.001	<0.001	0.003	0.010

Means in the same column with different superscripts differ at p < 0.05. SEM, standard error of the mean; IVADDM, *in vitro* apparent degradable dry matter; IVTDDM, *in vitro* true degradable dry matter; PF24, partitioning factor at 24 h; MM, microbial mass.

### 3.3. Dry matter and fiber fraction degradability

The fungi strain × incubation time interaction had a significant (p < 0.001) effect on nutrient degradability. Similarly, the main effect of fungi strain (p < 0.05) and incubation time (linear, p < 0.01) influenced nutrient digestibility (Table 3). P2 at weeks 6 and 8 had higher (p < 0.001) DMD, while P2 and P3 at week 2 had the lowest DMD values. Higher NDFD values were observed with P3 at weeks 0 and 2, while the lowest values were observed in P1 at weeks 4 and 8. Higher (p < 0.001) ADFD and ADLD values were recorded in P2 at week 8. Lower HEMD was observed in P2 at week 8, while P2 at week 6 had a higher CELD value. Notably, DMD, ADFD, and CELD increased as the incubation time increased but NDFD, ADLD, and HEMD decreased with increasing incubation time.

### 3.4. Volatile fatty acids production

The fungi strain × incubation time interaction, as well as the main effect of strain, had significant (p < 0.05) effects on C_2_, C_3_, C_2_:C_3_, and iso-C_5_ proportions (Table 4). However, incubation time did not (p > 0.05) affect the total and molar proportion of individual VFA. Higher C_2_ proportion (p = 0.035) and C_2_:C_3_ ratios (p = 0.026) were recorded in P3 at weeks 0 and 2, and lower values were observed in P2 at week 6. Conversely, a higher (p = 0.001) C_3_ proportion was observed in P2 at week 6, while lower values were observed in P3 at weeks 0 and 2.

### 3.5. Estimated parameters

Significant (p < 0.05) fungi strain × incubation period interaction was observed in all estimated parameters (Table 5). Similarly, the main effect of fungi strain and incubation time (linear effects) had a significant (p < 0.01) effect on all estimated parameters. Higher (p < 0.05) DMI, DDM, RFV, TDN, RFQ, DCP, DE, ME, NEM, and NEL values were observed with P1 at week 8, while higher (p < 0.01) GE was observed in P1 at week 6. Lowest DMI, DDM, RFV, TDN, RFQ, DE, ME, NEM, and NEL values were observed with P2 at week 0 and P3 at week 2. The lowest DCP (p = 0.011) was observed with P2 at week 0, and the lowest GE values were observed in P1 at week 8, followed by P2 at week 8.

### 3.6. Correlation between parameters

The correlation analysis revealed several significant relationships between nutrient content and fermentation characteristics of *Pleurotus*-treated corn stover (Tables 6 and 7). DM had weak positive correlations with CP (r = 0.27, p < 0.05), ADF (r = 0.44, p < 0.01), ADL (r = 0.30, p < 0.01), CEL (r = 0.29, p < 0.01), undegraded residuals (r = 0.29, p < 0.01), and PF_24_ (r = 0.31, p < 0.01). OM exhibited a linear relationship (p < 0.01) with NDF (r = 0.40), HEM (r = 0.44), CEL (r = 0.60), undegraded residuals (r = 0.37), and MM (r = 0.27). An inverse correlation (p < 0.01) was observed between NSC and NDF (r = −0.88), ADF (r = −0.76), ADL (r = −0.61), HEM (r = −0.76), CEL (r = −0.43), and undegraded residuals (r = −0.67). Additionally, DMD had a positive linear correlation (p < 0.01) with NSC (r = 0.46) and GP (r = 0.73) and an inverse relationship with NDF (r = −0.57), ADF (r = −0.40), HEM (r = −0.57), and undegraded residuals (r = −0.89). Propionate had a weak linear correlation with GP (r = 0.24, p < 0.05) and was inversely related with undegraded residuals (r = −0.31, p < 0.01). Butyrate demonstrated a linear correlation with GP (r = 0.26; p < 0.01).

**Table 3.** Dry matter and fiber digestibility (% DM) of corn stover treated with *Pleurotus ostreatus* P1 and P3 isolates and *P*. *pulmonarius* P2 isolate.

Strain	Week	DMD	NDFD	ADFD	ADLD	HEMD	CELD
P1	0	22.7^defgh^	82.9^bcd^	63.8^h^	25.5^bc^	19.1^a^	38.2^h^
	2	23.4d^efg^	79.0^gh^	67.0^def^	22.4^cdef^	12.0^cde^	44.6^ef^
	4	24.7d^ef^	77.7^h^	68.4^cde^	23.1^cde^	9.32^ef^	45.3^de^
	6	21.0^fgh^	81.1^defg^	66.4^efg^	13.8^i^	14.7^bc^	52.6^b^
	8	26.2^cd^	77.6^h^	68.8^cd^	27.1^b^	8.8^efg^	41.8^fg^
P2	0	19.3^gh^	84.4^ab^	64.6^gh^	26.6^b^	19.9^a^	38.0^h^
	2	18.1^h^	84.1^abc^	66.2^fg^	22.0^def^	17.9^ab^	44.3^ef^
	4	29.3^bc^	79.4^fgh^	69.7^c^	19.3^fg^	9.70^def^	50.4^bc^
	6	36.2^a^	78.9^gh^	72.1^b^	15.3^hi^	6.81^fg^	56.7^a^
	8	33.0^ab^	81.5^defg^	77.5^a^	30.9^a^	5.06^g^	46.6^de^
P3	0	21.5^efgh^	85.6^a^	63.9^h^	24.9^bcd^	21.6^a^	39.1^gh^
	2	18.2^h^	84.7^ab^	65.1^fgh^	21.3^efg^	19.6^a^	43.9^ef^
	4	20.8^fgh^	82.1^bcde^	72.9^b^	20.3^efg^	9.21^ef^	52.6^b^
	6	23.4^defg^	81.8^cdef^	73.3^b^	20.2^efg^	8.51^efg^	53.1^b^
	8	25.8^cde^	79.8^efgh^	66.4^efg^	18.1^gh^	13.4^cd^	48.3^cd^
SEM		0.64	0.33	0.44	0.54	0.63	0.64
p-value							
Strain		<0.001	<0.001	<0.001	0.026	0.007	<0.001
Week							
Linear		<0.001	<0.001	<0.001	0.004	<0.001	<0.001
Quadratic		0.450	0.005	<0.001	<0.001	<0.001	<0.001
Strain × Week		<0.001	<0.001	<0.001	<0.001	<0.001	<0.001

Means in the same column with different superscripts differ at p < 0.05. SEM, standard error of the mean; DMD, dry matter disappearance; NDFD, neutral detergent fiber disappearance; ADFD, acid detergent fiber disappearance; ADLD, acid detergent lignin disappearance; HEMD, hemicellulose disappearance; CELD, cellulose disappearance.

**Table 4.** Total (mmol) and individual volatile fatty acid (%) of corn stover treated with *Pleurotus ostreatus* P1 and P3 isolates and *P*. *pulmonarius* P2 isolate.

Strain	Week	VFA	C_2_	C_3_	C_2_:C_3_	C_4_	C_5_	Iso-C_4_	Iso-C_5_
P1	0	34.4	72.7^abc^	19.0b^cde^	3.85^abc^	7.21	0.14	0.46	0.52^abc^
	2	36.0	70.2^bc^	20.2^abcd^	3.54^bc^	8.19	0.17	0.56	0.64^abc^
	4	34.5	71.5^abc^	18.9^bcde^	3.84^abc^	8.06	0.19	0.63	0.67^a^
	6	30.4	73.5^abc^	17.5^de^	4.20^abc^	7.62	0.18	0.60	0.61^abc^
	8	31.1	73.3^abc^	17.4^de^	4.22^abc^	7.85	0.17	0.59	0.64^abc^
P2	0	32.0	72.6^abc^	19.0^bcde^	3.84^abc^	7.30	0.15	0.48	0.50^c^
	2	30.8	72.6^abc^	18.0^cde^	4.05^abc^	8.02	0.18	0.58	0.62^abc^
	4	34.1	70.4^bc^	20.7^abc^	3.41^bc^	7.74	0.14	0.48	0.56^abc^
	6	39.4	69.0^c^	22.2^a^	3.11^c^	7.61	0.13	0.45	0.54^abc^
	8	34.4	69.7^bc^	21.1^ab^	3.31^bc^	7.89	0.16	0.52	0.66^ab^
P3	0	39.8	75.2^a^	17.0^e^	4.88^ab^	6.70	0.15	0.48	0.51^bc^
	2	39.6	75.2^a^	16.8^e^	5.38^a^	6.88	0.15	0.48	0.50^c^
	4	31.3	74.0^ab^	17.9^cde^	4.15^abc^	7.01	0.15	0.48	0.49^c^
	6	31.7	72.5^abc^	18.9^bcde^	3.86^abc^	7.34	0.17	0.56	0.56^abc^
	8	33.7	73.6^ab^	18.1^cde^	4.17^abc^	7.07	0.16	0.53	0.55^abc^
SEM		0.746	0.40	0.30	0.134	0.100	0.001	0.002	0.001
p-value									
Strain		0.546	0.002	0.001	0.006	0.521	0.210	0.193	0.006
Week									
Linear		0.226	0.179	0.987	0.126	0.989	0.652	0.532	0.260
Quadratic		0.856	0.714	0.479	0.591	0.372	0.731	0.555	0.557
Strain × Week		0.210	0.035	0.001	0.026	0.634	0.679	0.692	0.040

Means in the same column with different superscripts differ at p < 0.05. SEM, standard error of the mean; VFA, total volatile fatty acids; C_2_, acetate; C_3_, propionate; C_2_:C_3_, acetate: propionate ratio; C_4_, butyrate; C_5_, valerate; Iso-C_4_, isobutyrate; Iso-C_5_, isovalerate.

**Table 5.** Estimated dry matter intake and nutritive and energy values of corn stover treated with *Pleurotus ostreatus* P1 and P3 isolates and *P*. *pulmonarius* P2 isolate.

Strain	Week	DMI	DDM	RFV	TDN	RFQ	DCP	GE	DE	ME	NEM	NEL
P1	0	1.48^h^	46.9^cd^	53.7^f^	34.8^cd^	41.8^e^	0.97^c^	4.13^abc^	1.53^cd^	1.26^cd^	0.72cd	0.84^cd^
	2	1.75^de^	50.5^b^	68.5^c^	40.7^b^	58.0^bc^	2.10^bc^	4.18^abc^	1.79^b^	1.47^b^	0.89^b^	0.99^b^
	4	1.80^c^	50.8^b^	71.1b^c^	41.3^b^	60.6^b^	2.81^ab^	4.01^de^	1.82^b^	1.49^b^	0.91^b^	1.00^b^
	6	1.78^cd^	50.5^b^	69.8^c^	40.8^b^	59.1^bc^	2.22^bc^	4.23^a^	1.80^b^	1.48^b^	0.89^b^	0.99^b^
	8	1.96^a^	54.0^a^	82.0^a^	46.6^a^	74.3^a^	3.67^a^	3.99^e^	2.06^a^	1.69^a^	1.06^a^	1.13^a^
P2	0	1.47^h^	46.3^d^	52.6^fg^	33.7^d^	40.2^e^	1.17^c^	4.13^abc^	1.49^d^	1.22^d^	0.69^d^	0.81^d^
	2	1.60^g^	47.9^c^	59.3^e^	36.5^c^	47.3^d^	1.70^bc^	4.11^abcd^	1.61^c^	1.32^c^	0.77^c^	0.88^c^
	4	1.78^cd^	50.2^b^	69.4^c^	40.3^b^	58.4^bc^	2.35^abc^	4.08^bcde^	1.78^b^	1.46^b^	0.88^b^	0.97^b^
	6	1.87^b^	50.4^b^	73.1^b^	40.6^b^	61.8^b^	2.12^bc^	4.18^abc^	1.79^b^	1.47^b^	0.89^b^	0.98^b^
	8	1.87^b^	48.0^c^	69.4^c^	36.7^c^	55.6^c^	2.90^ab^	3.85^f^	1.62^c^	1.33^c^	0.77^c^	0.89^c^
P3	0	1.49^h^	46.2^d^	53.6^f^	33.6^d^	40.9^e^	2.27^abc^	4.06^cde^	1.48^d^	1.22^d^	0.69^d^	0.81^d^
	2	1.47^h^	44.3^e^	50.6^g^	30.4^e^	36.4^f^	2.75^ab^	4.13^abc^	1.34^d^	1.10^d^	0.59^d^	0.73^d^
	4	1.69^f^	46.7^cd^	61.1^de^	34.5^cd^	47.3^d^	2.75^ab^	4.20^ab^	1.52^cd^	1.25^cd^	0.71^cd^	0.83^cd^
	6	1.71^ef^	47.4^cd^	63.0^d^	35.6^cd^	49.6^d^	2.69^ab^	4.14^abc^	1.57^cd^	1.29^cd^	0.74^cd^	0.86^cd^
	8	1.74^e^	51.3^b^	69.4^c^	42.1^b^	59.7^b^	2.60^ab^	4.18^abc^	1.86^b^	1.52^b^	0.93^b^	1.02^b^
SEM		0.024	0.38	1.34	0.64	1.54	0.134	0.017	0.020	0.017	0.013	0.011
p-value												
Strain		<0.001	<0.001	<0.001	<0.001	<0.001	0.018	0.016	<0.001	<0.001	<0.001	<0.001
Week												
Linear		<0.001	<0.001	<0.001	<0.001	<0.001	<0.001	0.005	<0.001	<0.001	<0.001	<0.001
Quadratic		<0.001	0.583	<0.001	0.584	0.003	0.195	<0.001	0.583	0.577	0.597	0.595
Strain × Week		<0.001	<0.001	<0.001	<0.001	<0.001	0.011	<0.001	<0.001	<0.001	<0.001	<0.001

Means in the same column with different superscripts differ at p < 0.05. SEM, standard error of the mean; DMI, dry matter intake; DDM, digestible dry matter; RFV, relative forage value; TDN, total digestible nutrient; RFQ, relative forage quality; DCP, digestible crude protein; GE, gross energy.

**Table 6.** Pearson correlation between nutrient contents and fermentation characteristics of corn stover treated with *Pleurotus*.

Variables	DM	OM	CP	NSC	NDF	ADF	ADL	HEM	CEL
DM	1								
OM	−0.01	1							
CP	0.27*	−0.36**	1						
NSC	−0.26*	0.03	0.25*	1					
NDF	0.19	0.40**	−0.49**	−0.88**	1				
ADF	0.44**	0.21*	−0.37**	−0.76**	0.81**	1			
ADL	0.30**	−0.54**	0.07	−0.61**	0.35**	0.46**	1		
HEM	−0.04	0.44**	−0.47**	−0.76**	0.91**	0.49**	0.19	1	
CEL	0.29*	0.60**	−0.45**	−0.43**	0.67**	0.80**	0.16	0.42**	1
GP	−0.33**	−0.28*	0.05	0.33**	−0.40**	−0.37**	0.19	−0.33**	−0.28*
Undegraded	0.29*	0.37**	−0.25*	−0.67**	0.76**	0.64**	0.29*	0.67**	0.52**
PF24	0.31**	0.09	0.12	0.02	−0.01	0.08	0.04	−0.07	0.06
MM	0.1	0.27*	0.11*	0.19*	−0.07	−0.14	−0.10	0	−0.09

DM, dry matter; OM, organic matter; CP, crude protein; EE, ether extract; NSC, non-structural carbohydrate; NDF, neutral detergent fiber; ADF, acid detergent fiber; ADL, acid detergent lignin; HEM, hemicellulose; CEL, cellulose; GP, gas production; PF24, partitioning factor at 24 h; MM, microbial mass.

**Table 7.** Pearson correlation between nutrient contents and *in vitro* fermentation products of corn stover treated with *Pleurotus*.

Variables	DM	OM	CP	NSC	NDF	ADF	ADL	HEM	CELL	GP	Undegraded	PF24	MM
DMD	−0.09	−0.38**	0.19	0.46**	−0.57**	−0.40**	−0.17*	−0.57**	−0.32**	0.73**	−0.89**	−0.26**	0.03
NDFD	0.29*	0.16	−0.31**	−0.67**	0.70**	0.71**	0.32**	0.54**	0.57**	−0.07	0.62**	−0.30**	−0.27**
ADFD	0.22*	−0.46**	0.34**	0.44**	−0.61**	−0.15	0.08	−0.81**	−0.21*	0.12	−0.50**	0.24*	−0.05
ADLD	−0.04	−0.63**	0.05	−0.45**	0.17	0.16	0.72**	0.13	−0.30**	−0.22*	0.05	0.24*	−0.10
HEMD	0.01	0.35**	−0.39**	−0.68**	0.78**	0.48**	0.16	0.82**	0.43**	−0.12	0.66**	−0.31**	−0.14
CELLD	0.18	0.21*	0.19	0.68**	−0.56**	−0.24*	−0.55**	−0.66**	0.11	0.27**	−0.38**	−0.04	0.06
VFA	0.08	−0.01	−0.13	−0.12	0.11	0.13	0.03	0.08	0.12	−0.04	0.02	0.03	−0.08
C2	−0.03	0.20*	−0.07	−0.07	0.16	−0.02	−0.07	0.25*	0.03	0.02	0.14	−0.14	0.03
C3	−0.18	0.08	−0.03	0.17	−0.11	−0.14	−0.17	−0.06	−0.05	0.24*	−0.31**	−0.09	0.14
C2:C3	0.13	0.13	−0.09	−0.22*	0.25*	0.13	0.06	0.28*	0.1	−0.16	0.34**	−0.04	−0.05
C4	−0.27*	0.06	0.02	0.17	−0.12	−0.22*	−0.11	−0.02	−0.17	0.26**	−0.19	−0.23	0.17
C5	−0.12	0.01	0.09	0.07	−0.07	−0.14	−0.01	0	−0.15	−0.38**	0.05	0.35**	0.33**
Iso–C4	−0.14	0.03	0.1	0.13	−0.11	−0.18	−0.06	−0.02	−0.17	−0.36**	0.01	0.36**	0.34**
Iso–C5	−0.27*	−0.09	0.12	0.21	−0.23*	−0.29	−0.06	−0.13	−0.29**	0.06	−0.22	0	0.26

Footnotes as in Tables 4 and 6.

## 4. Discussion

### 4.1. Chemical composition

The nutrient profile of feed resources is essential for making informed decisions regarding their inclusion in diet formulations for various categories of farm animals across different production and physiological stages. The SSF of corn stover with WRF strains had profound effects on nutrient composition. Fungi mycelia secrete compounds into their substrates, which help to release bound nutrients and affect the chemical composition of substrates [27],[28]. Furthermore, incubation time had a linear effect on nutrient concentrations, confirming the ability of the fungal strains to degrade the incubated substrate through exogenous secretions, as well as the different rates of growth between different species and isolates [29]. The CP content was at its lowest with P1 at week 0, but it reached its peak after 8 weeks of SSF, possibly due to the fungi's capacity to efficiently convert lignin-rich residues into fungal biomass over time. Fungi strains have the ability to capture excess N through aerobic fermentation and convert it into microbial protein [30]. The different CP concentrations between the three fungal species may be due to the different genetic backgrounds of the isolates. Olagunju et al. [27] observed a 58.5% increase in the CP content of *P. ostreatus*-treated corn stover. Increasing the CP content in poor nutritive value materials such as corn stover is recommended before feeding them to animals. This allows ruminal microbes to utilize the dietary CP for their growth and activity, ultimately meeting the animals' protein and amino acid requirements [31].

Similar to the CP trend, the NSC content was lowest in P3 at week 0 and highest in P2 at week 6, suggesting that fungal metabolism of the readily soluble carbohydrates occurred as the mycelia colonization progressed [30]. These results suggest an improvement in the nutritive value of corn stover after 6 weeks of incubation, as a higher concentration of NSC provides the ruminal microflora with more energy. Increasing the concentration of NSC validates the disruption of the carbohydrate–cell wall complex and the release of its soluble carbohydrates [32].

The highest NDF and ADF values were observed in P2 at week 0, while the highest CEL and HEM values were observed in P1 at week 0 of SSF. Conversely, the lowest NDF and ADF were observed in P1 at 8 weeks of SSF, possibly as a result of the breakdown of the lignocellulosic complex by the fungi strains over time [1],[15]. Although similar results were previously reported by Geib et al. [11] and Anotaenwere et al. [10], they did not observe a significant effect on ADL reduction due to the short SSF period (3 weeks). Most experiments found that WRF treatments decrease ADL concentration [10],[14],[15],[27].

### 4.2. In vitro gas and DM digestibility

Gas production is an indicator of the availability of fermentable carbohydrates for enteric fermentation in ruminants. It can be influenced by several factors, including diet composition, rumen microbiome composition, and metabolic activity, as well as the types and inclusion levels of feed additives [33],[34]. We expected fungi strain and incubation period to affect GP because they both affected the concentrations of nutrients in the incubated substrates. P2 at weeks 6 and 8 and P1 at week 4 produced more gases compared to P3 at weeks 2 and 4, which produced the lowest GP. The observed increases in GP at later weeks may be attributed to changes in nutrient concentration during ensiling [35]. As the ensiling process progressed, the concentration of soluble carbohydrates increased, providing an easily accessible energy source to support the growth and activities of the microbes [36]. This is consistent with Nayan et al. [37], who reported that a greater amount of gas produced in treated samples could be because of readily accessible fermentable carbohydrates, as the correlation analysis showed that GP was positively correlated with NSC (r = 0.33). Moreover, Benson et al. [28] reported that increased GP could be directly related to increased NSC concentrations after SSF of corn stover with *P. ostreatus*. Benson et al. [28] further stated that increased GP could indicate the availability of more nutrients for rumen microbes to use, signifying the improved nutritive value of treated corn stover. Recently, an experiment by Anotaenwere et al. [10] reported that GP was positively correlated with OM, CP, EE, and NSC, while it was negatively correlated with NDF, ADF, ADL, and HEM. In the present study, the correlation analysis showed that GP was negatively correlated with DM (r = −0.33), OM (r = −0.28), NDF (r = −0.40), ADF (r = −0.37), HEM (r = −0.33), and CEL (r = −0.28), and all of these decreased with WRF SSF treatments.

Fungal strain and incubation period affected IVADDM, IVTDDM, and PF_24_, confirming the differences between the evaluated strains in producing a variety of compounds with increasing incubation period. The highest IVADDM and IVTDDM were observed with P2 at weeks 6 and 8, indicating the ability of P2 to produce an array of exogenous compounds (e.g., ligninolytic enzymes). The concentrations of these compounds increased as the incubation time increased. The results of IVADDM and IVTDDM reflected in the amounts of the undegraded residues, where the highest amounts were observed in P3 and P2 at week 2 each, compared to P2 at weeks 6 and 8, which had the lowest undegraded values. A lower undegraded value is desirable because it simply means that a larger portion of the animal feed will be digested and utilized by the animal after undergoing ruminal fermentation. This result is consistent with Benson et al. [28], who reported that an increase in DM resulted in lower undegraded portions.

Lowering PF24 values in P1 at weeks 4 and 6 could be related to the increased GP with the same treatments. The PF_24_ represents the allocation of truly digested OM between microbial biomass and fermentation gases during the fermentation process. Consequently, the PF_24_ is mathematically inversely related to gas volume. The correlation analysis showed a strong negative correlation (p < 0.01) between GP and PF_24_ (r = −0.80). Increasing PF_24_ in P3 at weeks 4 and 6 is consistent with the results of Olagunju et al. [9] who reported an inverse relationship between *in vitro* GP and PF_24_. The correlation analysis showed that PF_24_ was correlated with GP (r = −0.80), MM (r = 0.27), DMD (r = −0.26), NDFD (r = −0.30), and HEMD (r = −0.31), and all of them increased or decreased with fungal treatments.

Microbial biomass synthesis has the potential to meet 70%–100% of the protein needs of ruminants [38]. P3 had the highest MM at week 8, while P2 had the lowest MM value at week 8. We expected the concentrations of NSC and CP to be the primary drivers of these results, but our correlation analysis revealed weak correlations between MM and CP (r = 0.11) and MM and NSC (r = 0.19), suggesting other factors, such as their degradability, and not their concentrations. We expected a strong correlation between CP and MM because higher CP contributes additional amino acids and peptides to the growth of ruminal microorganisms, increasing microbial biomass synthesis. We found a weak correlation (r = 0.27) between CP and MM (P < 0.05), which means that other factors, such as the amino acid profile, are more important in making MM than the concentration of CP. For ruminants, microbial mass is an excellent alternative source of proteins, and a high degree of carbon fixation in microbial cells can reduce fermentable carbon losses in the form of gases, especially CO_2_ and CH_4_ [24]. The present results are consistent with previous studies [39],[40], which reported an inverse relationship between *in vitro* GP and MM yield. A similar observation was also noted by Olagunju et al. [27].

### 4.3. Dry matter and fiber fraction degradability

The highest DMD observed in P2 at weeks 6 and 8 indicated that more nutrients would be utilized and readily available for farm animals. This desirable observation could be attributed to the delignification of P2 during SSF to release utilizable nutrients embedded in the corn stover. Moreover, it could be the result of a higher concentration of NSC, particularly starch [36],[41]. This is consistent with other studies [5],[15] that reported that the selective removal of lignin from rice straw by WRF (*P. ostreatus*) improved the ruminal degradation of carbohydrates. Additionally, Olagunju et al. [27] reported an increase in the DMD of *P. ostreatus*-treated corn stover after 24 h of *in vitro* rumen fermentation. The correlation analysis confirms that increasing NSC (r = 0.46) and reducing NDF concentration (r = −0.57), as well as HEM (r = −0.57), may be responsible for increasing DMD.

After *in vitro* rumen fermentation, P2 at week 6 had a higher CELD value than P2 at week 0, which had the lowest CELD value. The percentage differences for both ADFD and CELD were 21.5% and 49.2%, respectively. This result is consistent with the reports of Russell et al. [42], who stated that the higher digestibility of ADF and CEL in comparison with control samples indicated an improved nutrient utilization and energy availability for ruminants. The report further specified that increased CELD is associated with better animal energy utilization. In another study, Zhao et al. [43] reported a 54.3% decrease in HEM content, but the CEL content increased from 32.3% to 34.1%. Results from the current study align with their report, showing a greater percentage of HEMD (217.2%) in the SSF corn stover, as the percentage difference between the lowest and highest, and a percentage increase of 49.2% in CELD. Except for P3 at week 8, the near-uniform decreases in treatment values observed in NDFD were consistent with Zhao et al. [43], who reported a significant removal of HEM compared to CEL. This might be because WRF consumed the HEM in the corn stover during the SSF process, during which mycelia and the corn stover interacted. CELD was negatively correlated with the concentrations of NDF (r = −0.56), ADL (r = −0.55), and HEM (r = −0.66), with no correlation with CEL content, indicating that these nutrients may be the reason for the consistent CELD results. Interestingly, there was a positive correlation (r = 0.68) between the concentration of NSC and CELD, suggesting that the fungi mycelia used NSC as an energy source to produce the enzymes responsible for CELD [44].

Except for P1 and P2 at week 8, ADLD decreased after *in vitro* rumen fermentation compared to untreated substrates. The percentage difference reported for ADLD was 123.9%. This result is consistent with Anike et al. [12], who reported that the trend of lignin degradation was invariably similar across co-substrates and increased with time. The report further stated that there was no significant difference in lignin degradation between corn stover and peanut shell substrates after 60 days of SSF. However, lignin degradation was highest after 120 d of SSF on all substrates, irrespective of substrate composition. This further aligns with the results of Godlewska and Ciepiela [45], who stated that AD is inversely related to forage digestibility and that high lignin content is associated with reduced total feed digestibility. Our correlation analysis confirmed this assumption since ADL was negatively correlated with CEL digestibility (r = −0.55).

### 4.4. Volatile fatty acid production

Volatile fatty acids play a pivotal role in the rumen fermentation process, serving as a key energy source for ruminants [46]. It was anticipated that variations in OM, NSC, and fiber concentrations, along with differences in nutrient digestibility among the feeds, would result in different concentrations of total and individual VFAs; however, this was not observed. The correlation analysis did not show any correlation with nutrient concentration or degradability. However, P3 at week 2 recorded increased C_2_ proportions, C_2_:C_3_ ratios, and the lowest C_3_ proportions after *in vitro* rumen fermentation. An inverse relationship observed between C_2_ content and the duration of incubation suggests a shift in the rumen fermentation pathway due to the presence of more NSC in the treated samples. More specifically, P2 at weeks 6 and 8 of incubation had the lowest C_2_ and highest C_3_ concentrations as compared to other strains and incubation periods. This showed that substrates fermented by P2 favored C_3_ production after *in vitro* rumen fermentation. The shift in the fermentation pathway could be detrimental to the activities of methanogenic bacteria and could result in the reduction of methane and energy loss in ruminants during digestion. The decrease in C_2_ and increase in C_3_ are consistent with a previous study by Anike et al. [12], who stated that rapidly fermentable carbohydrates could result in an increased production of C_3_. Olagunju et al. [9] reported a significant decrease in C_2_ and an increase in C_3_, signifying a favored rumen fermentation pathway with WRF-treated substrates. This implies that the OM, CEL, and HEM contents present in the SSF corn stover substrates were efficiently utilized [27]. Compared with the controls, the proportion of Iso-C_5_ was higher in the treated substrates, with the highest value observed in P1 at week 4, except for P3 at weeks 2 and 4, which had the lowest proportion. A previous study by Dewhurst et al. [47] suggested that excess ruminal degradable protein in the diet could be the cause of higher molar proportions of iso-C_5_ in the diet.

### 4.5. Estimated parameters

Fungi strain and incubation period had impacts on all estimated parameters except DCP. Similar to the CP concentration in different treatments, P2 had the lowest DCP at week 0; however, at the end of the *in vitro* rumen fermentation, P1 recorded the highest estimated parameters, while P2 and P3 had the lowest values at weeks 0 and 2, respectively. Such improvements could be related to the reduction of structural carbohydrates by the fungi strains during the bioconversion process. Higher levels of digestibility and DMI are essential to improving production performance in any livestock sector [24]. The results showed that corn stover after SSF with P2 at weeks 6 and 8 could elevate the utilization of corn stover in livestock production by increasing the DMI of corn stover, which is typically known to have a lower DMI due to the presence of lignin [48]. In addition, the observed decrease in ADF and NDF contents across all treatments, except for P3 at week 2 across both variables, could have resulted in the increase in DMI. This is consistent with previous studies [49],[50], which reported an increase in the daily DMI of fungi-treated straw in an *in situ* experiment.

The significant differences observed in the GE of treated corn stover could reflect OM utilization in treated groups for fungal growth [12],[50]. Though the observed GE values and patterns were not consistent, it is worth noting the assumption that GE and ash have an inverse relationship [51]. This is very evident in both P1 and P2, where lower GE values had correspondingly higher ash content values.

The commercial-scale application of SSF-treated corn stover holds significant promise for improving ruminant nutrition, reducing feed costs, and promoting sustainability in livestock production. SSF improved the digestibility and protein content of corn stover, enabling the partial replacement of conventional feed ingredients, which can lower overall production costs. While there are initial investments required for microbial cultures, fermentation infrastructure, and drying or ensiling facilities, large-scale processing optimization can improve economic feasibility. Integrating SSF-treated stover into feeding programs may boost feed efficiency and animal performance, helping to offset processing costs and deliver a favorable return on investment. Additionally, using this abundant agricultural byproduct reduces waste, cuts reliance on land-intensive feed crops, and supports sustainability initiatives, such as carbon credit programs. However, for widespread adoption to occur, challenges related to processing logistics, storage stability, and farmer acceptance must be addressed. These hurdles could be mitigated with government incentives, cooperative feed manufacturing, and supportive policies for circular agriculture.

## 5. Conclusions

Among the strains tested, results indicate that *P. pulmonarius* requires 6 weeks to decompose and produce the highest possible grade of treated corn stover. *P. pulmonarius* (P2) at week 6 attained the highest DMD, CEL content, gas volume production, *in vitro* truly digestible dry matter, and propionate concentration and the lowest acetate concentration. Treatment of corn stover with *P. pulmonarius* (P2) for 6 weeks resulted in a nutrient-rich feed suitable for ruminant production. The use of WRF-treated corn stover in ruminant feeding is currently a hot topic of research. However, recent studies, including this one, indicate lower DMD for WRF-treated corn stover compared to conventional hay and silage. Therefore, further research is needed to optimize the SSF conditions and use of WRF species that will yield greater feed values for fermented corn stover. Additionally, WRF-treated corn stover contains many complex biomolecules from the fungi used in SSF. The roles of such molecules in the overall health and productivity of ruminants are not captured in our study, nor in many others already published on this subject. Further studies focusing on parameters beyond traditional feed value measurements and *in vivo* studies that allow for a comprehensive evaluation of animal health and productivity are recommended before WRF-treated biomass can be adequately compared with the feed values of hay and silage.

## Use of AI tools declaration

The authors declare they have not used Artificial Intelligence (AI) tools in the creation of this article.
